# Application of high-frequency ultrasound to assess facial skin thickness in association with gender, age, and BMI in healthy adults

**DOI:** 10.1186/s12880-022-00839-w

**Published:** 2022-06-16

**Authors:** Ying Meng, Lan Feng, Jiali Shan, Zixu Yuan, Lin Jin

**Affiliations:** Department of Ultrasound, Jiading District Central Hospital Affiliated Shanghai University of Medicine & Health Sciences, No. 1 Chengbei Road, Jiading District, Shanghai, 201800 China

**Keywords:** Ultrasound, Age, Skin thickness, Epidermis, Dermis

## Abstract

**Background and objective:**

High-frequency ultrasound plays an extremely important role in normal skin measurement, skin disease diagnosis, and aesthetic medicine. This study aimed to estimate the epidermal and dermal thicknesses at eight different facial sites in healthy adults using high-frequency ultrasound, and to evaluate the correlation of epidermal and dermal thicknesses with age and body mass index (BMI).

**Methods:**

Facial skin assessment was performed on 118 participants using high-frequency ultrasound. The epidermal and dermal thicknesses of forehead, glabella, temple, eyelid, nasal dorsum, zygoma, submandibular, and neck were measured. The correlation of the epidermal and dermal thicknesses with age and BMI was analyzed by the linear correlation analysis.

**Results:**

The epidermal and dermal thicknesses in men were significantly higher than those in women (*P* < 0.05), except for the thicknesses of zygomatic epidermis and neck dermis. The dermal thickness on zygoma and submandibular in young women was significantly higher than in middle age and old women (*P* < 0.05). Overall, with the increase of age, the thickness of facial skin decreased in women, mainly in the forehead, glabella, zygoma, and submandibular. In women, the epidermal and dermal thicknesses of neck were correlated with BMI (*r* = 0.392, 0.241, *P* < 0.05, respectively). However, in men, the epidermal and dermal thicknesses were correlated with age only in zygoma dermis (*r* = − 0.327, *P* < 0.05), while there was no correlation between the epidermal and dermal thicknesses and BMI.

**Conclusion:**

Gender, age, and BMI had significant effects on the epidermal and dermal thicknesses at different facial sites.

## Background

Skin provides an effective barrier between the organism and the environment, preventing the invasion of pathogens and fending off chemical and physical assaults. Long-term exposure to solar ultraviolet radiation is the primary factor of extrinsic skin aging. With the gradual improvement of living standards and the development of medical beauty technology, the consumer acceptance of aesthetic medicine consumption has gradually been boosted in China [1]. Facial skin, as one of the important indicators of "beauty", has noticeably attracted aesthetic surgeons’ and dermatologists’ attention [2]. Skin thickness is one of the important indices of facial aging [3]. More accurate quantitative evaluation of skin rather than simple subjective evaluation is also of great importance in aesthetic medicine.

In recent years, with the increased frequency of ultrasonic probes, especially probes with frequency ≥ 20 MHz, different layers of skin can be clearly distinguished, and it is widely used in dermatology [4]. At present, the most commonly used probe frequency in clinic is 20–25 MHz, which can clearly show the epidermis, dermis, subcutaneous soft tissue, and the junction between them.

The present study aimed to measure the epidermal and dermal thicknesses at eight different facial regions using ultrasound scanning with 24 MHz ultrasonic probes, and to assess their correlations with age, gender, body mass index (BMI), and facial site.

## Methods

### Study design and population

The present study enrolled 118 healthy adults who were admitted to Jiading District Central Hospital Affiliated to Shanghai University of Medicine & Health Sciences (Shanghai, China). These participants underwent high-frequency ultrasound scan. Clinical investigations were performed according to the Declaration of Helsinki. Furthermore, all participants signed the informed consent form prior to enrollment. The study protocol was approved by the Ethics Committee of Jiading District Central Hospital Affiliated to Shanghai University of Medicine & Health Sciences (Approval No. 2019-B-15). Participants were asked to avoid using any skincare products or make-up within 1 day before testing.

### Inclusion criteria

The inclusion criteria were as follows: Participants who aged ≥ 18 years old; Participants who voluntarily participated in this study and signed the informed consent form; Patients with no systemic diseases and/or other diseases that might cause changes in skin thickness; The facial regions of participants were not treated with other methods before involvement in the study.

### Exclusion criteria

The exclusion criteria were as follows: Participants with a history of facial skin disorders, or those who received corticosteroid drugs; Participants with dermal lesions and/or crusts or marked keratinization in measurement sites; Participants who were strongly exposed to sun in previous 3 months, or whose jobs required excessive sun exposure and/or hard physical activities; Participants who were unable to fully participate in examinations.

### Ultrasound examinations

**Fig. 1:**
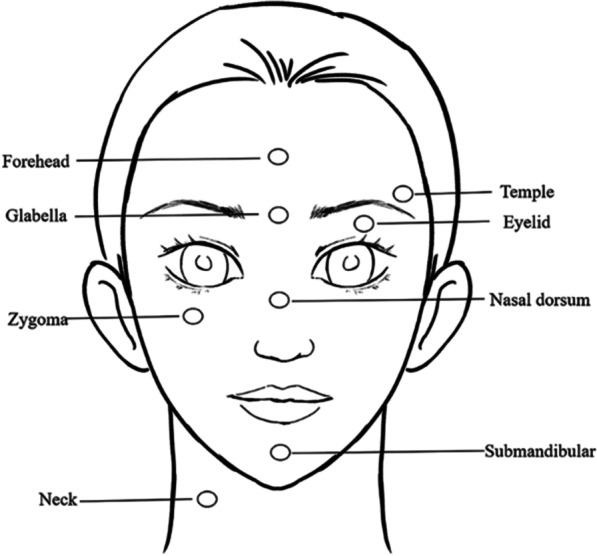
Sketch map of different facial skin thicknesses detected by high-frequency ultrasound

Eight facial sites were considered for ultrasonic assessment (forehead, glabella, temple, eyelid, nasal dorsum, zygoma, submandibular, and neck) (Fig. 1). These facial sites were identified for measurement by the anatomical landmarks. The thicknesses of epidermis and dermis in these facial sites was measured respectively.

The examinations were obtained at the same time of day in order to avoid a diurnal variation in dermal oedema. The volunteers underwent the examination in a supine position with his hands on both sides and his shoulders relaxed and breathed calmly.

The ultrasound device (Canon, Aplio i800, Japan) equipped with a 24 MHz linear probe was used. The test site were coated with a moderate thickness of ultrasounic gel layer to fill the space between the probe and the skin surface to enhance test sensitivity and focuse at the most superficial cutaneous layers. Furthermore, the probe was placed on the skin surface vertically and gently to avoid compression of the anatomical structures. All images were obtained using the same setting and were captured until a clear epidermal and dermal layer was observed. Then, the thicknesses of epidermis and dermis at each facial site were measured for three times, and the average value was calculated.

The distance between the thin strip and strong echo at the entrance of the epidermis was recorded as the epidermal thickness. The dermal thickness included the wide medium echo area between the back of the epidermis and the hypoechogenic subcutaneous fat layer.

Two observers were recruited for assessment of interobserver variability. One observer was designated for all 118 patients (observer A, with 20 years of experience in diagnostic ultrasound), while the other observer (observers B, with 10 years of experience in diagnostic ultrasound) measured the thicknesses of epidermis and dermis for the assigned 18 volunteers, respectively, to assess interobserver variability. Both observers were experienced sonographers and were unaware of each patient’s clinical information or status.

### Baseline measurements

Data of a comprehensive health and lifestyle questionnaire for study participants were collected with their consent. Data, including age, sex, history of facial allergy and facial exfoliation, smoking status, and alcohol consumption were obtained using a self-administered questionnaire. Weight and height were measured by nurses following standardized protocols, and BMI was calculated as weight/height^2^ (kg/m^2^).

### Statistical analysis

Statistical analysis was performed using SPSS 23.0 software (IBM, Armonk, NY, USA). The data were expressed as numbers and percentages by category for qualitative variables, and as mean ± standard deviation, or as median and interquartile range for abnormally distributed quantitative variables. One-way analysis of variance (ANOVA) or independent-samples t-test was applied to compare intergroup differences in epidermal and dermal thicknesses of participants. Interobserver variability was examined by intraclass correlation coefficient (ICC).

## Results

### Baseline characteristics

A total of 118 participants were included in the present study. Participants’ baseline characteristics are presented in Table 1. There were 49 (41.53%) male participants, with an average age of 46.45 ± 12.80 years old. There were 69 (58.47%) female participants, with an average age of 44.06 ± 11.10 years old. According to the World Health Organization (WHO) classification of age, participants’ age was divided into three levels: 20–44, 45–59, and ≥ 60 years old. In this study, 62 (52.54%), 42 (35.59%), and 14 (11.86%) participants aged 20–44, 45–59, and ≥ 60 years old, respectively.

**Table 1:** Baseline characteristics of the three groups

Variables	18–44 years (n = 62)	45–59 years (n = 42)	≥ 60 years (n = 14)	*F/χ*^2^	*P* value
*Sex n (%)*				3.608	0.165
Male	25 (40.32%)	15 (35.71%)	9 (64.29%)		
Female	37 (59.68%)	27 (64.29%)	5 (35.71%)		
Smoking	15 (24.19%)	9 (21.43%)	4 (28.57%)	0.312	0.856
Alcohol use (%)	33 (53.23%)	16 (38.10%)	4 (28.57%)	4.032	0.133
Allergy (%)	9 (14.52%)	11 (26.19%)	1 (7.14%)	3.565	0.168
Height (cm)	164.37 ± 7.28	161.17 ± 7.41^*^	165.71 ± 7.21^#^	3.201	0.044
Weight (Kg)	63.10 ± 11.17	60.29 ± 10.64	66.21 ± 10.21	1.782	0.173
BMI (kg/m^2^)	23.23 ± 2.89	23.10 ± 2.92	24.03 ± 2.74	0.563	0.571

Data are means ± SD or n[%]. BMI, body mass index; SD, standard deviation

Compared with the group 18–44 years old

*P < 0.05; Compared with the group 45–59 years old

^#^*P* < 0.05

### Interobserver agreement

As measured by the two observers, the mean values of epidermal thickness of the whole face were 0.18 mm and 0.18 mm, respectively, while the mean values of dermal thickness of the whole face were 0.98 mm and 0.97 mm, respectively. The pre-consensus measurements had a good ICC of 0.81 for epidermal thickness (95% confidence interval (CI) 0.56, 0.92) and an excellent ICC of 0.83 for dermal thickness (95% CI 0.60, 0.93).

The mean epidermal and dermal thicknesses of the whole face achieved by two observers were comparable, and the ICC for the interobserver variability was over 0.8, suggesting a good agreement between the two observers.

### Ultrasound measurements of epidermis and dermis thickness

Multivariate analysis of variance showed that gender had a significant effect on the thicknesses of facial dermis and epidermis of normal adults (*P* < 0.05), while age had no significant effect on the thicknesses of facial dermis and epidermis of normal adults.

Except for the thicknesses of zygomatic epidermis and neck dermis, the thicknesses of epidermis and dermis in other facial sites in men were significantly thicker than those in women, and the difference was statistically significant (*P* < 0.05) (Table 2). Forehead had the highest epidermal and dermal thicknesses in male participants, while the dermal thickness was the highest on neck in female participants.

**Table 2:** Comparison of epidermal and dermal thickness at different facial sites

Facial region	Male (n = 49)	Female (n = 69)	*t*	*P* value
*Epidermis (mm)*				
Forehead	0.22 ± 0.03	0.20 ± 0.03	3.356	0.001
Glabella	0.21 ± 0.03	0.19 ± 0.02	2.961	0.004
Temple	0.19 ± 0.02	0.18 ± 0.02	2.476	0.015
Eyelid	0.18 ± 0.03	0.17 ± 0.02	3.156	0.002
Nasal dorsum	0.20 ± 0.03	0.19 ± 0.02	2.356	0.020
Zygoma	0.19 ± 0.03	0.20 ± 0.18	− 0.322	0.748
Submandibular	0.21 ± 0.09	0.18 ± 0.03	2.220	0.028
Neck	0.19 ± 0.03	0.17 ± 0.03	3.926	< 0.001
*Dermis (mm)*				
Forehead	1.17 ± 0.28	0.86 ± 0.16	7.515	< 0.001
Glabella	1.14 ± 0.30	0.90 ± 0.23	4.972	< 0.001
Temple	1.06 ± 0.20	0.80 ± 0.19	7.272	< 0.001
Eyelid	0.67 ± 0.14	0.58 ± 0.11	4.192	< 0.001
Nasal dorsum	1.03 ± 0.16	0.93 ± 0.25	2.604	0.010
Zygoma	1.14 ± 0.21	0.87 ± 0.35	4.938	< 0.001
Submandibular	1.02 ± 0.24	0.91 ± 0.34	1.990	0.049
Neck	0.98 ± 0.22	0.99 ± 0.20	− 0.117	0.907

**Fig. 2:**
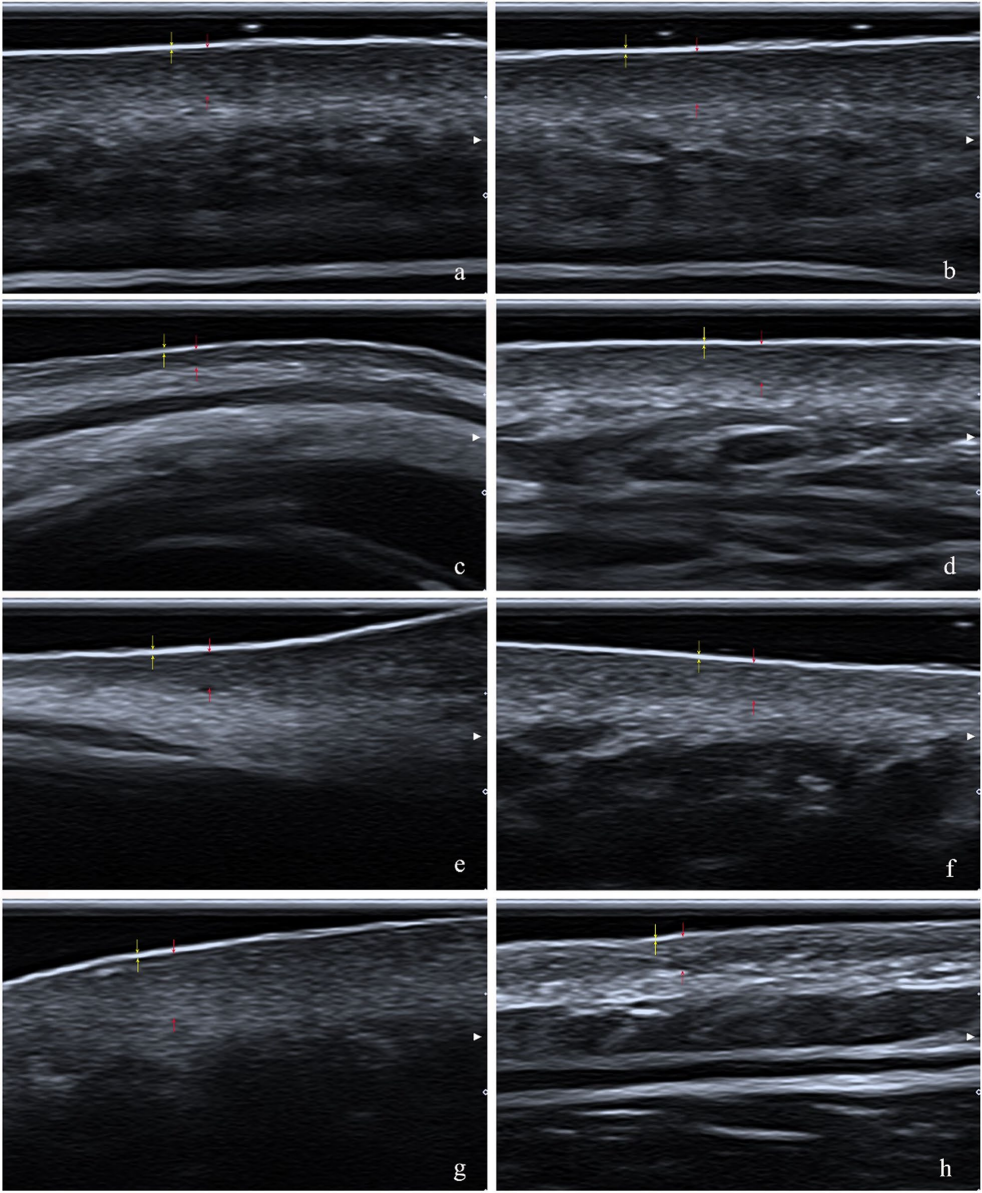
Ultrasound images of dermis and epidermis at different facial sites. Yellow arrow is epidermal thickness; Red arrow is dermal thickness. **a** forehead; **b** glabella; **c** temple; **d** eyelid; **e** nasal dorsum; **f** zygoma; **g** submandibular; **h** neck

Comparison of epidermal and dermal thicknesses in female participants between age-based groups (20–44, 45–59, and over 60 years old) showed that the dermal thickness was significantly thicker in zygoma and submandibular in the group of 20–44 years old (*P* < 0.05) (Fig. 2). In male participants, the thicknesses of epidermis and dermis were lower in the group of over 60 years old in all facial sites (statistical significance was only found in neck epidermis) (Table 3).

**Table 3:** Comparison of epidermal thickness ultrasound measures among the three groups

Facial region	Male					Female				
	18–44 years (n = 25)	45–59 years (n = 15)	≥ 60 years (n = 9)	*F*	*P* value	18–44 years (n = 37)	45–59 years (n = 27)	≥ 60 years (n = 5)	*F*	*P* value
*Epidermis (mm)*										
Forehead	0.23 ± 0.03	0.22 ± 0.03	0.20 ± 0.04	1.555	0.222	0.20 ± 0.04	0.20 ± 0.02	0.19 ± 0.02	0.145	0.865
Glabella	0.21 ± .025	0.21 ± 0.04	0.19 ± 0.03	1.670	0.199	0.20 ± 0.02	0.19 ± 0.02	0.20 ± 0.01	0.894	0.414
Temple	0.19 ± 0.02	0.19 ± 0.02	0.18 ± 0.02	1.500	0.234	0.18 ± 0.02	0.18 ± 0.03	0.17 ± 0.01	0.566	0.571
Eyelid	0.19 ± 0.03	0.18 ± 0.02	0.16 ± 0.02	2.575	0.087	0.17 ± 0.02	0.16 ± 0.03	0.17 ± 0.02	0.937	0.397
Nasal dorsum	0.20 ± 0.03	0.21 ± 0.03	0.19 ± 0.02	1.809	0.175	0.19 ± 0.02	0.20 ± 0.02	0.18 ± 0.01	0.567	0.570
Zygoma	0.19 ± 0.03	0.19 ± 0.02	0.18 ± 0.02	0.912	0.409	0.18 ± 0.02	0.23 ± 0.28	0.18 ± 0.04	0.752	0.476
Submandibular	0.22 ± 0.12	0.20 ± 0.02	0.18 ± 0.02	0.927	0.403	0.18 ± 0.03	0.19 ± 0.02	0.18 ± 0.01	0.688	0.506
Neck	0.19 ± 0.03	0.20 ± 0.03	0.17 ± 0.02^#^	3.650	0.034	0.17 ± 0.03	0.17 ± 0.03	0.18 ± 0.03	0.406	0.668
*Dermis (mm)*										
Forehead	1.22 ± 0.30	1.12 ± 0.27	1.12 ± 0.23	0.753	0.477	0.90 ± 0.14	0.83 ± 0.17	0.77 ± 0.17	2.598	0.082
Glabella	1.16 ± 0.33	1.15 ± 0.29	1.05 ± 0.20	0.510	0.604	0.95 ± 0.24	0.85 ± 0.23	0.75 ± 0.06	2.548	0.086
Temple	1.07 ± 0.22	1.04 ± 0.20	1.07 ± 0.18	0.178	0.838	0.82 ± 0.18	0.78 ± 0.19	0.71 ± 0.27	1.007	0.371
Eyelid	0.68 ± 0.15	0.65 ± 0.11	0.69 ± 0.17	0.183	0.834	0.56 ± 0.10	0.59 ± 0.11	0.63 ± 0.16	0.869	0.424
Nasal dorsum	0.98 ± 0.17	1.08 ± 0.13	1.09 ± 0.17	2.431	0.099	0.93 ± 0.24	0.90 ± 0.27	1.01 ± 0.20	0.393	0.677
Zygoma	1.21 ± 0.21	1.08 ± 0.18	1.07 ± 0.19	2.790	0.072	0.97 ± 0.33	0.78 ± 0.36^*^	0.63 ± 0.20^*^	3.853	0.026
Submandibular	1.07 ± 0.23	0.98 ± 0.22	0.96 ± 0.31	1.117	0.336	1.00 ± 0.29	0.84 ± 0.39	0.60 ± 0.26^*^	4.112	0.021
Neck	0.96 ± 0.19	1.04 ± 0.28	0.95 ± 0.19	0.730	0.488	0.96 ± 0.16	1.02 ± 0.24	1.03 ± 0.18	0.838	0.437

Compared with the 18-44 years old group , *P < 0.05; Compared with the 45-59 years old group, ^#^*P* < 0.05

### Correlation of epidermis and dermis thickness with age and BMI

In female participants, the dermal thickness of forehead, glabella, zygoma, and submaxilla was correlated with age (*r* = − 0.284, − 0.361, − 0.403, − 0.342, *P* < 0.05, respectively). The epidermal and dermal thicknesses of neck were correlated with BMI (*r* = 0.392, 0.241, *P* < 0.05, respectively) (Table 4).

**Table 4:** Results of univariate the association of Female epidermal and dermal thicknesses with age and BMI

	Age	BMI
*Epidermis*		
Forehead	− 0.069	0.052
Glabella	− 0.022	0.188
Temple	− 0.072	0.097
Eyelid	− 0.126	0.229
Nasal dorsum	− 0.016	0.045
Zygoma	− 0.012	0.070
Submandibular	0.170	0.122
Neck	0.128	0.392^**^
*Dermis*		
Forehead	− 0.284*	0.037
Glabella	− 0.361**	− 0.255^*^
Temple	− 0.209	− 0.081
Eyelid	0.147	0.087
Nasal dorsum	0.065	− 0.056
Zygoma	− 0.403**	− 0.198
Submandibular	− 0.342**	− 0.110
Neck	0.199	0.241^*^

*P < 0.05, **P < 0.001

In male participants, the epidermal and dermal thicknesses were correlated with age only in zygoma (r = − 0.327, *P* < 0.05), while there was no correlation between the epidermal and dermal thicknesses and BMI.

## Discussion

The present study aimed to evaluate the epidermal and dermal thicknesses in different facial regions using ultrasound scanning with 24 MHz ultrasonic probes to assess their correlations with age, gender, BMI, and facial site. We found that there were differences in the epidermal and dermal thicknesses of different genders and different facial sites in adults. A gradual decrease in dermal thickness with age in forehead, glabella, zygoma, and submandibular dermis was more significant in women than in men. With the increase of BMI, the thickness of facial skin changes in women, mainly in the glabella, neck dermis, and neck epidermis.

The skin consists of epidermis, dermis, and subcutaneous tissue. Facial skin is affected by several external environmental factors for a long time, such as sunlight, temperature, and humidity, which is more obvious than that of other parts of the skin [5]. With the occurrence of skin aging, the three components of the skin have different degrees of degenerative changes, and the skin thickness also changes [6]. Delaying skin aging, especially the facial skin, has noticeably attracted dermatologists’ attention. Skin thickness is one of the important indicators of facial aging.

Ultrasound as a non-invasive diagnostic method has been used to determine the whole skin thickness in the field of dermatology [7]. At present, high-frequency ultrasound with the characteristics of being noninvasive, rapid, and economic has been gradually applied in dermatology [8].

The results of the present study showed that the epidermal and dermal thicknesses in men were higher than those in women, which were consistent with Seidenari et al.’s findings [9]. In our study, the difference was statistically significant, except for the thickness of zygomatic epidermis and neck dermis.

Our results related to skin thickness were in accordance with outcomes achieved by Escoffier et al. [10] and Shuster et al. [11], who demonstrated that the acoustical properties of skin vary in association with age and gender. In men, there was no significant correlation between the epidermal and dermal thicknesses of facial skin and age. However, in women, the dermal thickness of forehead, glabella, zygoma, and submandibular gradually decreased with age. A previous research showed that with the occurrence of skin aging, among the three components of the skin, the change of dermal thickness was the most obvious [6], which was consistent with our findings. The differences between men and women may be caused by the particularity of female aging. A study found that the variation of estrogen level may change the skin thickness by affecting the amount of collagen [12, 13]. Most women began to change their menstrual cycle from the age of about 40 years old, so as to start skin aging from 40 years old. After the age of 48 years old, the skin thickness decreases linearly with the increase of age, and the aging is significantly accelerated from 60 to 70 years old [14, 15].

The neck is also an important component of the facial appearance. With the increase of age, the aging of the neck can be manifested as skin relaxation, wrinkles or fat accumulation, especially in the inherent neck [16]. In the present study, we found that the epidermal and dermal thicknesses in women were both correlated with BMI, however, no statistical significance was found in men. This also showed that there were differences in skin aging and skin thickness between men and women. Van Mulder et al. [17] demonstrated that elevation of BMI was associated with the increased skin thickness. However, only the epidermal and dermal thicknesses in women in our study were found to be correlated with BMI, potentially due to different facial sites. Our findings highly confirmed a positive relationship between skin thickness and BMI, while no remarkable influence of BMI at the facial sites was found.

## Limitations

The present study has several limitations. First, we did not assess the variations of epidermal and dermal thicknesses according to skin type, race, and other external environmental factors (e.g., sun exposure). Second, we did not evaluate the skin density, echogenicity, elasticity, and blood perfusion data of the epidermal and dermal layers. Thus, further study should be conducted to eliminate the above-mentioned shortcomings and to confirm our findings.

## Conclusions

In conclusion, evaluation of epidermal and dermal thicknesses using high-frequency ultrasound scanning is clinically advantageous. This indicates that high-frequency ultrasound can be broadly applied in dermatology and aesthetic medicine worldwide. The findings of our study suggested important differences in the thicknesses of skin epidermis and dermis in association with facial sites, gender, BMI, and age.

## Data Availability

The datasets used in the manuscript are available from the corresponding author upon reasonable request.
